# Age- and Gender-Related Mean Hearing Threshold in a Highly-Screened Population: The Korean National Health and Nutrition Examination Survey 2010–2012

**DOI:** 10.1371/journal.pone.0150783

**Published:** 2016-03-07

**Authors:** Yun Hwi Park, Seung-Ho Shin, Sung Wan Byun, Ju Yeon Kim

**Affiliations:** Department of Otorhinolaryngology, Ewha Womans University, School of Medicine, Seoul, Republic of Korea; IRCCS Istituto Auxologico Italiano, ITALY

## Abstract

**Background:**

In evaluating hearing disability in medicolegal work, the apportionment of age- and gender-related sensorineural hearing loss should be considered as a prior factor, especially for the elderly. However, in the literature written in the English language no studies have reported on the age- and gender-related mean hearing threshold for the South Korean population.

**Objective:**

This study aimed to identify the mean hearing thresholds in the South Korean population to establish reference data and to identify the age- and gender-related characteristics.

**Methods:**

This study is based on the Korea National Health and Nutrition Examination Survey (KNHANES) 2010–2012, which was conducted by the Korean government, the data of which was disclosed to the public. A total of 15,606 participants (unweighted) representing 33,011,778 Koreans (weighted) with normal tympanic membrane and no history of regular or occupational noise exposure were selected and analyzed in this study. The relationship between the hearing threshold level and frequency, age, and gender was investigated and analyzed in a highly-screened population by considering the sample weights of a complex survey design.

**Results:**

A gender ratio difference was found between the unweighted and the weighted designs: male:female, 41.0%: 59.0% (unweighted, participants) vs. 47.2%:52.8% (weighted, representing population). As age increased, the hearing threshold increased for all frequencies. Hearing thresholds of 3 kHz, 4 kHz, and 6 kHz showed a statistical difference between both genders for people older than 30, with the 4 kHz frequency showing the largest difference. This paper presents details about the mean hearing threshold based on age and gender.

**Conclusions:**

The data from KNHANES 2010–2012 showed gender differences at hearing thresholds of 3 kHz, 4 kHz, and 6 kHz in a highly-screened population. The most significant gender difference in relation to hearing threshold was observed at 4 kHz. The hearing thresholds at all of the tested frequencies worsened with increasing age. The mean hearing thresholds suggested in this study will be useful for the formulation of healthcare-related hearing policies and used as reference data for disability ratings for hearing loss due to various causes.

## Introduction

When court-ordered hearing examination is requested due to car accidents or work-related injuries, the age and gender of the patient are very important when determining the allocation of causation among the multiple factors that caused or significantly contributed to the injury or disease, and the resulting impairment. If a victim has no record of having undergone a hearing test prior to the car accident or work-related injury, the hearing threshold should be estimated. For example, in medicolegal cases, it might be difficult to consider if the pre-accident hearing threshold of a 70-year-old man is similar to that of a 20-year-old woman. Only a few reports have provided reference data for age- and gender-related hearing threshold levels for Koreans, and the studies that did had limitations in that their study population was uncertain [1] or the study sample suffered from selection bias [2]. For example, the data were collected from the health check-ups of healthy examinees [2] or from patients at a single institution [3, 4]. Recently, some articles have been published about hearing loss based on the Korean National Health and Nutrition Examination Survey (KNHANES) 2010–2012, which was conducted by the Korea Centers for Disease Control and Prevention. However, no study has reported on mean hearing thresholds for the Korean population using a complex survey design to analyze the data. When a complex design is ignored during statistical analysis, it results in biased estimates and overstated significance levels [5–7].

The KNHANES 2010–2012 was a complex, stratified, multistage, probability-cluster survey of a representative sample of the non-institutionalized civilian population in South Korea. Therefore, it provides reliable audiological data for stratified samples representing the entire Korean population. This present study aims to extract and objectively analyze the hearing data and to show the age- and gender-related authentic mean hearing threshold.

## Subjects and Methods

### Study Design (Complex Survey Design), Population, and Data Collection

The KNHANES is an ongoing cross-sectional survey of the civilian non-institutionalized population of South Korea. Many field survey teams performed interviews and physical and laboratory examinations. Those teams included otolaryngologists, ophthalmologists, and nurse examiners with mobile examination units.

The KNHANES participants were not randomly sampled. Since the survey was designed using a complex, stratified, multistage probability-sampling model based on the National Census Data, individual participants were not equally representative of the Korean population. The KNHANES website provides survey datasets that include information regarding the survey location, strata by age, sex, and various other factors, and the sample weight for each participant. In all of the analyses, survey sample weights were used, which were calculated by taking the sampling rate, response rate, and age/sex proportions of the reference population into account in order to provide representative estimates of the Korean civilian population. Previous articles have described the KNHANES methodology in detail [5, 8–11].

A total of 23,621 individuals (10,611 males and 13,010 females), representing 47,761,044 individuals (23,884,864 males and 23,876,180 females) in South Korea participated in the survey from July 2010 to December 2012. Among them, pure tone audiometry data were acquired from 8,147 male participants representing 19,826,055 Korean men and 10,435 female participants representing 19,689,497 women, whose ages ranged from 12 to 85.

### Selection of Subjects

To determine the mean hearing thresholds of the Korean population not affected by factors that are hazardous to hearing, including middle ear disease or noise exposure, better ears were selected and then the following exclusion criteria were established: participants with abnormal tympanic membrane on otoscopy or a history of regular or occupational noise exposure were excluded. To determine which ear (right or left) was better in individual participants, six-tone average thresholds (0.5, 1, 2, 3, 4, and 6 Hz), four-tone averages (0.5, 1, 2, and 3 kHz), and thresholds at 1 kHz, 2 kHz, 0.5 kHz, 3 kHz, 4 kHz, and 6 kHz, in both ears, were compared one-by-one. For example, if the six-tone average for the right ear is smaller than the left ear, the right ear is the better ear regardless of all the other thresholds. If the six-tone averages for both ears are the same, the four-tone averages for both ears are compared. **Table 1**shows the detailed exclusion procedures; 83.5% (weighted) of the representative population were included in this study.

**Table 1:** Selection of Participants with Normal Tympanic Membrane and No History of Noise Exposure.

	Participants (Unweighted, n = 18,582)		Representative Population (Weighted, n = 39,515,552)	
Exclusion criteria	No	Yes	No	Yes
Abnormal ear drum (A)	17,476 (94.0%)	1,106 (6.0%)	37,457,444 (94.8%)	2,058,108 (5.2%)
History of noise exposure (B)	16,590 (89.3%)	1,992 (10.7%)	34,805,691 (88.1%)	4,709,861 (11.9%)
(A) or (B)	15,606 (84.0%)	2,976 (16.0%)	33,011,778 (83.5%)	6,503,774 (16.5%)

### Audiometric Measure

Pure-tone audiometric testing was performed with a GSI SA-203 audiometer (Entomed; Lena Nodin, Sweden). Testing was conducted in a sound-treated booth inside the mobile examination center reserved for KNHANES. Otolaryngologists, who had been trained to operate the audiometer, provided instructions to participants and obtained the recordings. All audiometric testing was done under the supervision of an otologist. Only air conduction thresholds were measured. Supra-auricular headphones were used in the sound-treated booth. The otolaryngologist provided basic instructions to the participant regarding the automated hearing test. Automated testing was programmed according to a modified Hughson-Westlake procedure with appropriate masking. The lowest level at which the subject responded to 50% of the pure tone was set as the threshold. The automated hearing test involving air-conducted pure-tone stimuli showed good test-retest reliability and validity comparable to the manual pure-tone audio test [12, 13]. Participants responded by pushing a button when they heard a tone, and the results were automatically recorded [9]. The following frequencies were tested: 0.5 kHz, 1 kHz, 2 kHz, 3 kHz, 4 kHz, and 6 kHz. A retest threshold for 1 kHz was obtained to confirm threshold measurement consistency. Unless the difference between the two thresholds at 1 kHz was less than 5 dB, another audiogram was automatically repeated until that difference was less than 5 dB. To compare the hearing thresholds, the four-tone average (0.5 kHz, 1 kHz, 2 kHz, and 3 kHz) was calculated. The four-tone average is recommended by the American Academy of Otolaryngology-Head and Neck Surgery (AAO-HNS) [14].

### Ethics Statement

The KNHANES obtained a written informed consent from each participant prior to conducting the survey, and the Institutional Review Board of Ewha Womans University Mokdong Hospital approved this present research study (IRB No. ECT-201509-033).

### Statistical Analysis

Statistical analyses and graphing were performed using R software (version 3.2.1; The R Foundation, Vienna, Austria, URL http://www.R-project.org). The statistical values, including mean hearing thresholds and frequency averages, were calculated with the '*survey*' package from the R repository sites. The package was developed for analyses of complex survey samples and this made it possible to provide statistics taking sample weights and strata into consideration. Cross-tabulations, the calculations of *means*, and t-tests were performed using the '*svytables*', '*svymean*', and '*svyttest*' functions, respectively (in contrast to 'mean', '*xtabs*', and '*t*.*test*' functions for simple random sampling), according to gender and age. P values<0.05 were considered statistically significant.

**Fig 1:**
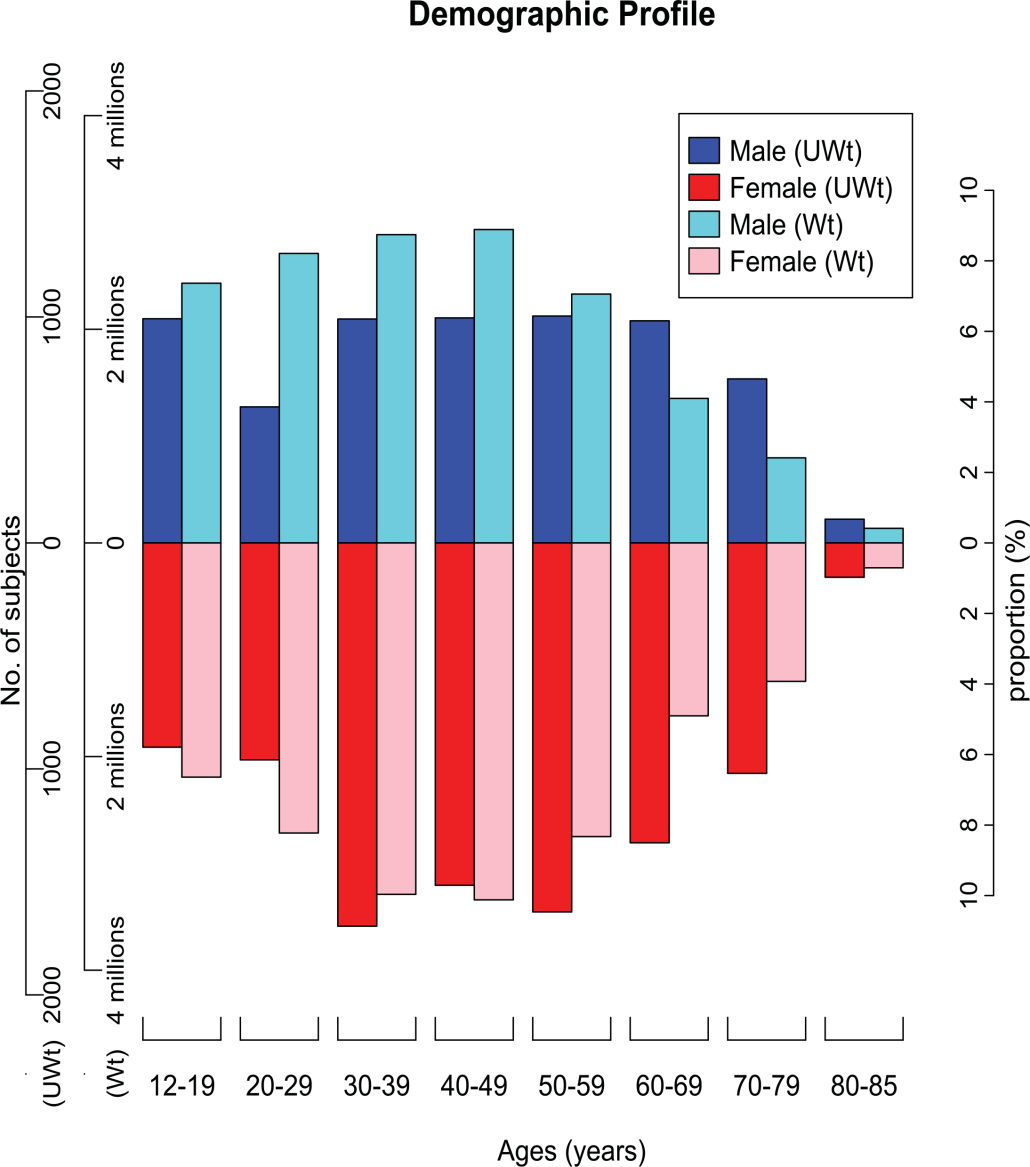
**Age- and gender-related distribution of participants (unweighted, the left 1st axis) and the corresponding population (weighted, the left 2nd axis)**. The blue and red columns show the distribution of 15,606 participants, and the cyan and pink columns represent 33,011,778 members of the Korean population, according to gender and age groups. The proportion of the participants is different from the proportion of the study population. This figure shows the difference between the occupation percentage of the unweighted and the weighted sample numbers, according to age group. If the unweighted design is used for analysis, male participants in their teens, twenties, thirties, forties, and fifties are under-sampled and participants in their sixties, seventies, and eighties are over-sampled. These ratio differences would affect the final outcome. In order to produce unbiased cross-section estimates for the entire Korean population, the specialized statistical technique of complex sampling design, considering survey sample weights and strata, should be utilized. UWt: Unweighted, Wt: Weighted.

**Fig 2:**
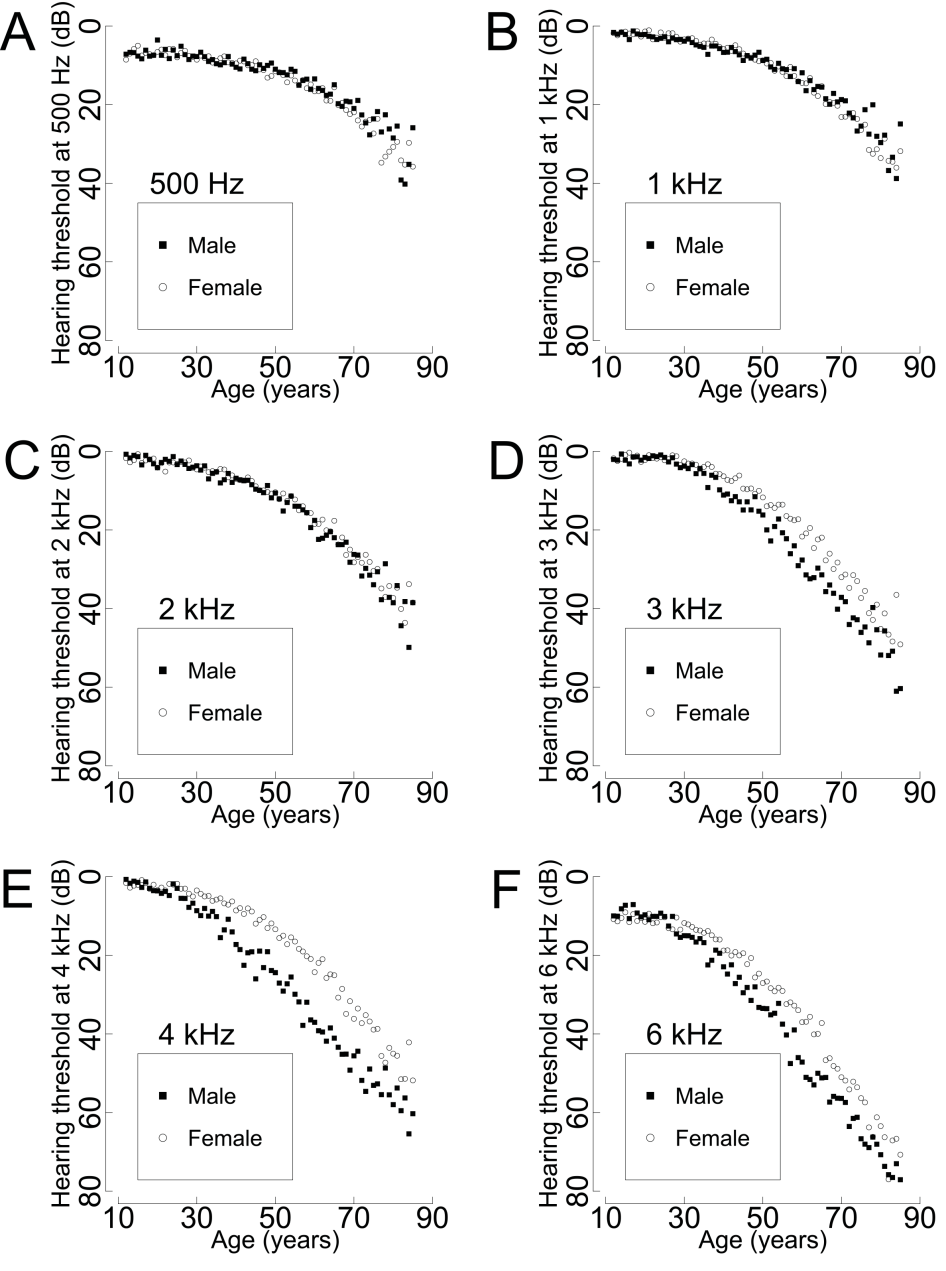
Gender difference of the mean hearing threshold in proportion to age at 0.5 kHz (A), 1 kHz (B), 2 kHz (C), 3 kHz (D), 4 kHz (E), and 6 kHz (F) frequencies (highly-screened population, N = 33,011,778 ears). At 0.5 kHz, 1 kHz, and 2 kHz, no difference in the mean threshold level was found between males and females. However, at 3 kHz, 4 kHz, and 6 kHz, the mean threshold levels for males are worse than the levels for females. Among the three frequencies, the most significant difference of the mean threshold level between males and females with the same age occurs at 4 kHz.

To find the distribution difference of the unweighted sample number and the representative population number by age and gender, a plot was made with triple Y axes (**Fig 1**). Mean hearing thresholds by age and gender according to frequency were calculated. Plots were made to discover the relationship between hearing threshold level and frequency, age, and gender (**Fig 2**).

**Fig 3:**
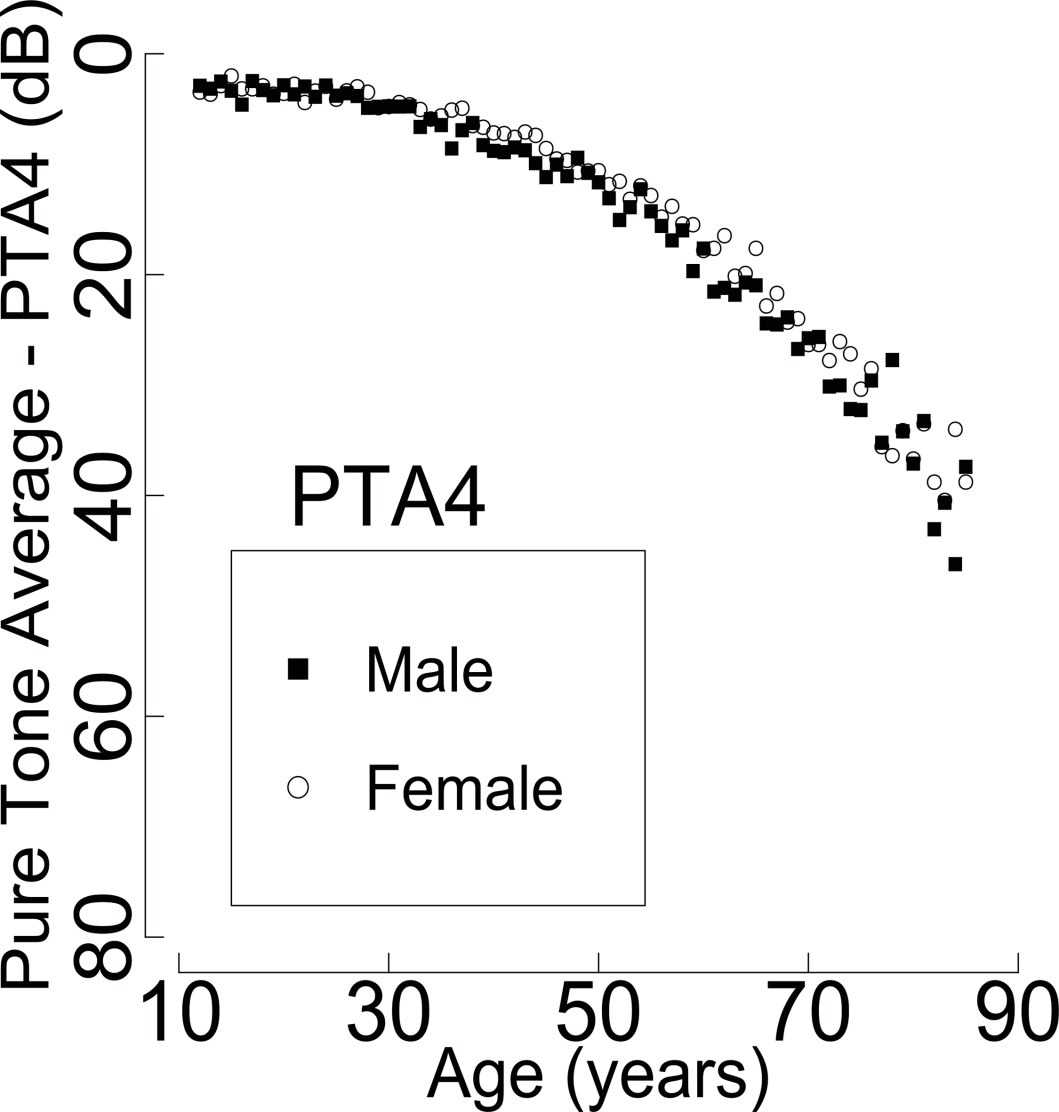
The four-tone average of 0.5 kHz, 1 kHz, 2 kHz, and 3 kHz (PTA4) (highly-screened population, N = 33,011,778 ears). Both genders showed similar four-tone average values. However, with increasing age, the gender difference of PTA4 tends to increase. PTA4: four-pure tone average.

**Fig 4:**
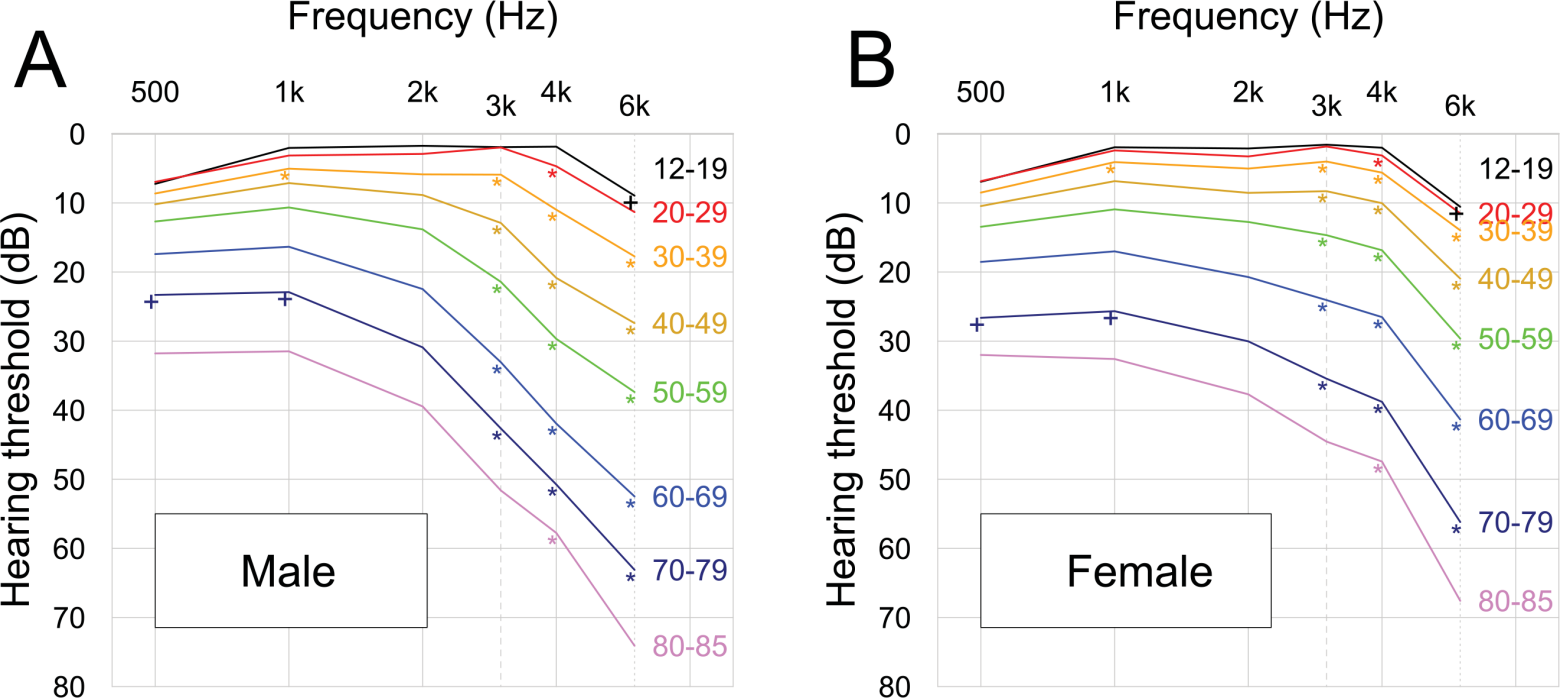
Mean audiograms for the Korean population as a function of gender, frequency, and age (highly-screened population, N = 33,011,778 ears). (A) Male; (B) Female. As age increased, the mean threshold at each frequency increased in both genders. *: The case in which the mean threshold is statistically significantly larger for males than for females. **†emT**he case in which the mean threshold is statistically significantly smaller for males than for females.

To compare the mean hearing threshold of both genders for each age group (12–19, 20–29, 30–39, 40–49, 50–59, 60–69, 70–79, and 80–85) at the six tested frequencies and the four-tone averages, multiple t-tests, adjusted by Bonferroni correction and based on the complex survey design, were performed. Plots were made to verify the gender difference of the four-tone averages (**Fig 3**). A mean pure tone audiogram based on age group is shown in **Fig 4**.

## Results

In the KNHANES 2010–2012 data, the number of highly-screened participants who underwent audiometric testing was 15,606, representing 33,011,778 Koreans (**Table 2**). The age of the participants ranged from 12 to 85. The mean age was 45.8±19.0 (unweighted) and 40.9±17.8 (weighted). Differences in the gender ratio were found between the unweighted and weighted designs: M:F, 41.0%: 59.0% (unweighted, participants) vs. 47.2%:52.8% (weighted, representing population) (**Fig 1**). In comparing the proportion of the unweighted and weighted groups by age (12–19, 20–29, 30–39, 40–49, and 50–59) in males, the unweighted groups were found to be under-sampled in contrast to the representative population. The unweighted groups by age (60–69, 70–79, and 80–85) in males were over-sampled in contrast to the weighted groups by the representative population.

**Table 2:** Demographic Data of Highly-Screened Participants in the Korea National Health and Nutrition Examination Survey (KNHANES), 2010–2012.

Processed as	Simple random sampling design (unweighted)	Complex survey design (weighted)
Total No. of participants	15,606	33,011,778
Sex		
Male	6,399 (41.0%)	15,575,206 (47.2%)
Female	9,207 (59.0%)	17,436,572 (52.8%)
Mean age ± SD (year-old)	45.8 ±19.0	40.9 ±17.8
Age range (years)	12–85	12–85

**Table 3**and **Fig 2**show the mean hearing threshold levels as a function of gender, frequency, and age. In addition, it provides the four-tone average threshold. Irrespective of gender and frequency, as the age of the participants increased, their hearing threshold increased. **Table 3**shows the statistical difference of the mean hearing threshold level by gender and age at 0.5 kHz, 1 kHz, 2 kHz, 3 kHz, 4 kHz, and 6 kHz. At frequencies of 0.5 kHz, 1 kHz, and 2 kHz, there was little statistical difference in the mean threshold level between males and females. However, for males older than 30 the mean threshold levels at 3 kHz, 4 kHz, and 6 kHz were significantly worse than the levels for females in the same age group. As seen in **Table 3**, the difference between the hearing thresholds for males and females at 3 kHz, 4 kHz, and 6 kHz began in the 30–39 age group and increased with increasing age until the 60–69 age group; however, the differences in these three hearing thresholds decreased in the 70–79 and 80–85 age groups for both genders. Among the latter three frequencies, the gender difference of the mean threshold level at 4 kHz was the most significant.

**Table 3:** Mean Hearing Threshold Level and Standard Deviation as a Function of Gender, Frequency, and Age Group in a Highly-Screened Population, and Comparison between Mean Thresholds of both Genders (M: F = 15,575,206: 17,436,572, weighted).

Age group	No. Participants	No. Representing population	Mean Hearing Thresholds (dB)						
			0.5 kHz	1 kHz	2 kHz	3 kHz	4 kHz	6 kHz	PTA4
	Male: Female	Male: Female	Male: Female	Male: Female	Male: Female	Male: Female	Male: Female	Male: Female	Male: Female
12–19	992: 904	2,431,315: 2,192,738	7.2: 6.9	2.0: 1.9	1.7: 2.1	1.9: 1.6	1.9: 2.0	**9.0: 10.6^†^**	3.2: 3.1
20–29	602: 960	2,709,851: 2,715,782	7.0: 6.8	3.1: 2.4	2.9: 3.3	2.0: 1.8	4.7: 3.1*	11.3: 11.5	3.7: 3.6
30–39	991: 1696	2,885,434: 3,289,781	8.6: 8.5	5.0: 4.1*	5.9: 5.0	5.9: 4.0*	11.0: 5.6*	17.7: 14.0*	6.4: 5.4*
40–49	996: 1515	2,933,533: 3,341,372	10.2: 10.5	7.1: 6.8	8.9: 8.5	12.9: 8.3*	20.9: 10.0*	27.4: 20.9*	9.8: 8.5*
50–59	1004: 1633	2,329,503: 2,749,101	12.7: 13.4	10.7: 10.9	13.8: 12.8	21.4: 14.7*	29.7: 16.8*	37.4: 29.6*	14.6: 13.0*
60–69	983: 1327	1,352,532: 1,618,758	17.4: 18.5	16.3: 17.0	22.5: 20.7	33.1: 24.1*	41.9: 26.5*	52.5: 41.3*	22.3: 20.1*
70–79	726: 1020	796,380: 1,295,721	**23.3: 26.6^†^**	**22.9: 25.7^†^**	30.9: 30.0	42.6: 35.4*	50.8: 38.8*	63.1: 56.2*	29.9: 29.4
80–85	105: 152	136,659: 233,319	31.8: 32.0	31.5: 32.6	39.5: 37.7	51.6: 44.5	57.8: 47.4*	74.10: 67.6	38.6: 36.7
			Standard Deviation (dB)						
12–19			±7.0: ±6.7	±6.7: ±5.8	±7.2: ±7.2	±7.9: ±7.9	±8.6: ±9.7	±10.0: ±12.4	±5.6: ±5.3
20–29			±7.6: ±8.2	±6.5: ±7.6	±7.7: ±9.9	±10.0: ±13.7	±12.9: ±17.7	±14.6: ±19.0	±6.1: ±7.3
30–39			±9.8: ±12.1	±10.2: ±13.3	±12.4: ±16.4	±17.7: ±20.7	±19.4: ±21.6	±21.1: ±22.0	±10.3: ±13.2
40–49			±13.7: ±17.9	±15.0: ±19.5	±18.4: ±20.4	±20.8: ±21.9	±20.8: ±20.5	±21.6: ±20.6	±14.4: ±17.8
50–59			±7.4: ±6.6	±6.8: ±6.3	±7.8: ±6.8	±8.2: ±7.3	±8.5: ±8.8	±10.4: ±10.8	±6.1: ±5.1
60–69			±7.3: ±8.0	±6.8: ±8.0	±6.8: ±8.8	±7.3: ±9.0	±8.9: ±10.4	±10.8: ±12.8	±5.2: ±6.8
70–79			±10.1: ±12.6	±10.2: ±13.0	±11.4: ±13.9	±12.2: ±14.8	±13.5: ±16.4	±16.9: ±19.9	±9.2: ±11.8
80–85			±15.8: ±16.0	±16.4: ±17.0	±16.5: ±16.4	±17.3: ±18.2	±18.0: ±19.7	±19.9: ±19.3	±14.7: ±15.0

**Bold**: Statistically significant (p<0.05, using Bonferroni correction)

* The case in which the mean threshold is larger for males than for females

^**†**^ The case in which the mean threshold is smaller for males than for females.

PTA4: Four-tone average threshold = (0.5 kHz + 1 kHz + 2 kHz + 3 kHz threshold)/4. Standard Deviations here are based on the distribution of participants (sample). Standard Errors for estimates of the population mean are provided in the supporting information.

The four-tone (0.5 kHz, 1 kHz, 2 kHz, and 3 kHz) averages are shown in **Fig 3**. The average between males and females was similar except for the 30–39, 40–49, 50–59, and 60–69 age groups.

**Fig 4**shows the mean audiograms for the Korean population as a function of gender, frequency, and age. As the age of the male or female participants increased, the hearing thresholds at all frequencies increased. The higher the frequencies, the greater the hearing loss.

## Discussion

This study used three years of pooled data: 2010, 2011, and 2012. The KNHANES 2010–2012 sampling weights ranged from 75.66 to 45,812.38 persons, and the median and mean values were 4,792.564 and 6470.565, respectively. The number of participants in 2010, 2011, and 2012 were 6,532, 6,302, and 5,817, respectively. The total Korean non-institutionalized population in 2010, 2011, and 2012 was 39,514,595, 40,324,566, and 38,978,241, respectively. The KNHANES guideline for data processing suggests that, in order to adjust sampling weights, the sample weights should be divided by 3; that is, the number of years of data pooled. Thus, the adjusted and pooled number representing the total Korean non-institutionalized population 2010–2012 was 39,605,801.

While several papers addressing hearing thresholds for the Korean population have been published, they have only focused on certain groups, including visitors at health screening centers, adolescents, male firefighters, the elderly, and residents of specific regions [1–4, 15]. Kim et al. presented hearing thresholds of 462 Korean adolescents who used a personal music player [3]. While no gender differences for the thresholds at all frequencies was found in the 12–19 age group in the present study, the authors did find gender differences at 4 kHz for the same age group. In addition, the three-tone average hearing thresholds in their study were much worse than the three-tone average thresholds in our study. It is assumed that the differences in the findings of these two studies resulted from the exclusion of participants with a history of noise exposure. Kang et al. compared the hearing threshold of male firefighters with hearing data from a screened and unscreened male population [15]. They found that male firefighters that were younger than 45 had worse hearing than the screened population.

It is difficult to directly compare their results with the results from our study, as they supplied median hearing thresholds. Bahng and Lee reported the hearing thresholds of 263 people whose ages ranged from 60 to 84 [1]. They found that the hearing thresholds gradually worsened with increasing age, and they showed gender differences in hearing thresholds at 1 kHz, 2 kHz, 3 kHz, 4 kHz, 6 kHz, and 8 kHz. Kim et al. reported on the hearing thresholds of 6,028 people who lived in three provinces in Korea [2]. They found that hearing loss was more prevalent in males than females over 65.

Kim et al. reported gender differences with hearing thresholds for 1,116 visitors at a health screening center that were otologically normal, and they reported no occupational or non-occupational noise exposure [4]. They found that males had a significantly greater rate of change in their hearing thresholds at 4 kHz and 8 kHz than females. The hearing threshold at 6 kHz was not checked in that study. While gender-related differences of mean threshold at each frequency were evaluated in the present study, the threshold change at each frequency by age was analyzed. In [4], the threshold change at 8 kHz was larger than the change at 4 kHz for males. However the gender difference of mean hearing threshold at 4 kHz by age groups was not stated. The study subjects were healthy clients who visited the health screening center. In that study, males who had a history of military service were excluded. Therefore, the sample size of males (N = 214) was remarkably smaller than the sample size (N = 902) of females. In contrast, in this present study, the study subjects were members of the general population and the sample size of males and females was similar. The authors provided mean thresholds and 95% confidence intervals by age groups. In this present study, while all the mean thresholds increased depending on age at all frequencies, they did not consistently increase in [4], probably because the sample size in certain age groups was too small.

Hoffman et al. reported hearing results from the US National Health and Nutrition Examination Survey (NHANES), 1999–2004 [16]. Their analysis was given in median (95% confidence intervals in an unscreened US population). In this present study, hearing thresholds in a highly-screened Korean population were obtained in mean value. To compare both studies, the data were divided in the same age groups. And then median thresholds at 0.5, 1, 2, 3, 4 and 6 kHz from our data were calculated. Across age and sex groups, difference of median thresholds between the NHANES 1999–2004 and KNHANES 2010–2012 at 0.5, 1, 2, 3, 4 and 6 kHz (8 kHz was not tested in this study) were less than 3 dB except three thresholds at 3 kHz for females aged 55 to 64 years and 6 kHz for females aged 35 to 44 and 45 to 54 years. So the median values of most frequencies in both studies were very similar. See the S2 Table of supporting files.

There have been controversies about gender differences in relation to hearing thresholds in the literature. Some studies have reported that there was no difference in hearing thresholds between males and females [17]. In contrast, other studies have found that hearing was worse in males than it was in females [1, 18–22]. In the present study, the remarkable finding is that 4 kHz is the frequency that showed the largest gender difference for mean hearing threshold (**Fig 2E**). **Fig 2D and 2F** shows that there is less of a gender difference for the mean threshold at 3 kHz and 6 kHz than at 4 kHz. The exclusion of subjects exposed to regular or occupational noise exposure was the minimum criteria used to eliminate the effect of noise on hearing thresholds. The real amount of exposure an individual has to noise cannot be investigated. It is assumed that the most remarkable reason for the gender difference at the 4 kHz threshold is due to socio-environmental factors, not biological differences; men are exposed to more noise than women. This hypothesis is supported by the tendency that gender difference for hearing thresholds decreased in retired people (>70 years).

**Fig 4**shows the mean pure-tone audiogram results by age and gender. As age increased, the mean threshold at each frequency increased in spite of gender. We thought that this pattern resulted from the aging effect. The hearing threshold for males was worse than it was for females. However, increased hearing thresholds at 6 kHz were noted, irrespective of age and gender, as compared to the other frequencies. This outcome might be due to the known calibration problem in the TDH-39 earphone of the GSI SA-203 audiometer, which could give rise to a roughly 5 dB increase at 6 kHz compared to the average thresholds for most other audiometric frequencies [16, 23].

The information from this present study, provided in **Table 3**and **Fig 4**, can be used as reference data to estimate the pre-accidental hearing threshold based on age and gender.

## Conclusions

Gender difference in mean hearing threshold was observed at the frequencies of 3 kHz, 4 kHz, and 6 kHz from the data obtained from the KNHANES 2010–2012. The most significant gender-related hearing threshold difference was observed at 4 kHz. Our results will be useful for formulating healthcare-related hearing policies, and they can be used as basic reference data for disability ratings for hearing loss due to various causes.

## Supporting Information

S1 Table Detailed numeric data for Figs 2 and 3. (CSV)

S2 Table Comparison between KNHANES 2010–2012 and NHANES 1999–2004. (DOCX)

S3 Table Detailed values for Table 3 including 95% confidence interval. (XLSX)
