# Supplementary material for: Age- and Gender-Related Mean Hearing Threshold in a Highly-Screened Population: The Korean National Health and Nutrition Examination Survey 2010–2012
Source: PLoS One. 2016 Mar 7;11(3):e0150783. doi: 10.1371/journal.pone.0150783 (PMC4780829; doi:10.1371/journal.pone.0150783)
Supplement: S2 Table — (DOCX) [file pone.0150783.s002.docx]

**S2_Dataset. Comparison between KNHANES 2010-2012 and NHANES 1999-2004.**

Median Hearing Thresholds between Korean National Health and Nutrition Examination Survey 2010–2012 (KNHANES) 2010-2012 and National Health and Nutrition Examination Survey (NHANES) 1999-2004 are shown according to frequency by age group and gender.

|  | Hearing Threshold Level (dB HL)  Age^*^ | | | | | | | |
| --- | --- | --- | --- | --- | --- | --- | --- | --- |
|  | 30 | | 40 | | 50 | | 60 | |
| Frequency (Hz) | KNHANES  2010-2012 | NHANES  1999-2004^#^ | KNHANES  2010-2012 | NHANES  1999-2004^#^ | KNHANES  2010-2012 | NHANES  1999-2004^#^ | KNHANES  2010-2012 | NHANES  1999-2004^#^ |
| Male |  |  |  |  |  |  |  |  |
| 500 | 6 | 7 | 9 | 8 | 10 | 10 | 14 | 11 |
| 1000 | 3 | 4 | 6 | 6 | 7 | 9 | 11 | 11 |
| 2000 | 4 | 4 | 5 | 6 | 10 | 10 | 14 | 14 |
| 3000 | 2 | 4 | 8 | 9 | 13 | 15 | 22 | 25 |
| 4000 | 5 | 7 | 12 | 13 | 21 | 22 | 33 | 35 |
| 6000 | 12 | 11 | 19 | 17 | 28 | 25 | 43 | 40 |
| Female |  |  |  |  |  |  |  |  |
| 500 | 7 | 7 | 10 | 7 | 11 | 9 | 14 | 13 |
| 1000 | 2 | 4 | 5 | 5 | 8 | 7 | 11 | 10 |
| 2000 | 3 | 4 | 5 | 5 | 10 | 7 | 14 | 11 |
| 3000 | 2 | 2 | 5 | 4 | 10 | 7 | 17 | 12 |
| 4000 | 5 | 4 | 6 | 7 | 11 | 10 | 19 | 16 |
| 6000 | 11 | 10 | 15 | 12 | 23 | 17 | 32 | 24 |

^*^Age is grouped in 10-yr intervals, that is, “30” represents ages 25–34 yrs, etc.

^#^ Hoffman HJ, Dobie RA, Ko CW, Themann CL, Murphy WJ. Americans hear as well or better today compared with 40 years ago: hearing threshold levels in the unscreened adult population of the United States, 1959-1962 and 1999-2004. Ear hear. 2010;31(6):725-34.
